# One-Step Soft Chemical Synthesis of Magnetite Nanoparticles under Inert Gas Atmosphere. Magnetic Properties and In Vitro Study

**DOI:** 10.3390/nano10081500

**Published:** 2020-07-30

**Authors:** Laura Madalina Cursaru, Roxana Mioara Piticescu, Dumitru Valentin Dragut, Robert Morel, Caroline Thébault, Marie Carrière, Hélène Joisten, Bernard Dieny

**Affiliations:** 1 National R & D Institute for Non-Ferrous and Rare Metals, INCDMNR-IMNR, 102 Biruintei blvd, 077145 Pantelimon, Ilfov, Romania; dragutv@imnr.ro; 2 Univ. Grenoble Alpes, CEA, CNRS, Spintec , 38000 Grenoble, France; robert.morel@cea.fr (R.M.); caro.thebault@gmail.com (C.T.); helene.joisten@cea.fr (H.J.); bernard.dieny@cea.fr (B.D.); 3 Univ. Grenoble Alpes, CEA, CNRS, IRIG-SyMMES, 38000 Grenoble, France; marie.carriere@cea.fr; 4 Univ. Grenoble Alpes, CEA, LETI, 38000 Grenoble, France

**Keywords:** magnetite, hydrodynamic diameter, pressure, temperature, hydrothermal synthesis, in vitro viability, mass magnetization, biomedical applications

## Abstract

Iron oxide nanoparticles have received remarkable attention in different applications. For biomedical applications, they need to possess suitable core size, acceptable hydrodynamic diameter, high saturation magnetization, and reduced toxicity. Our aim is to control the synthesis parameters of nanostructured iron oxides in order to obtain magnetite nanoparticles in a single step, in environmentally friendly conditions, under inert gas atmosphere. The physical–chemical, structural, magnetic, and biocompatible properties of magnetite prepared by hydrothermal method in different temperature and pressure conditions have been explored. Magnetite formation has been proved by Fourier-transform infrared spectroscopy and X-ray diffraction characterization. It has been found that crystallite size increases with pressure and temperature increase, while hydrodynamic diameter is influenced by temperature. Magnetic measurements indicated that the magnetic core of particles synthesized at high temperature is larger, in accordance with the crystallite size analysis. Particles synthesized at 100 °C have nearly identical magnetic moments, at 20 × 10^3^ μ_B_, corresponding to magnetic cores of 10–11 nm, while the particles synthesized at 200 °C show slightly higher magnetic moments (25 × 10^3^ μ_B_) and larger magnetic cores (13 nm). Viability test results revealed that the particles show only minor intrinsic toxicity, meaning that these particles could be suited for biomedical applications.

## 1. Introduction

Iron oxide based magnetic nanoparticles (IONPs) have received remarkable attention in a wide range of applications because of their unique physicochemical properties inherent to the nanoscale. Small size, high surface area, quantum confinement, and novel magnetic and optical effects open up new fields for application of iron oxides [1,2,3,4,5,6,7,8,9,10,11,12,13,14,15].

Conventional magnetic materials (ferromagnetic iron oxides) lose their permanent magnetization if they are studied or used as nanoparticles. Parallel to the practical uses of magnetic IONPs in electronics and catalysis, they have been widely considered for decades for magnetic hyperthermia goals and as contrast agents for magnetic resonance imaging (MRI) [16,17,18,19,20,21].

The IONPs show ability for the biomedical application; they need to possess suitable core size and monodispersity, acceptable hydrodynamic diameter, high saturation magnetization (Ms), high stability in biological fluid media, to be biocompatible and degradable with reduced toxicity over a large time scale, capable of clearance from the body post imaging [1,2,3,4,5,6,7,9,11]. Critical requirements for biomedical related fields are good values of magnetization and ability to form stable aqueous dispersions. Below approximately 30 nm size they start behaving superparamagnetically at room temperature. Larger values of susceptibility and magnetic moment with smaller values of coercivity and remanence make them most desirable for various biomedical applications [2,3,4,6,10,11].

The magnetic behavior of IONPs is crucial for their effectiveness in biomedical applications, partially based on their superparamagnetic properties. Therefore, IONPs often are labelled as SPIONs (superparamagnetic iron oxide nanoparticles). Superparamagnetism is a property occurring principally in nanoparticles which are single-domain and can be attributed to their size. The dependence of magnetic properties of SPIONs on the specific composition, structure, size, size distribution, and shape were the object of extensive studies throughout the years. The procedures for synthesis/surface coating/encapsulation, their effect on the physico-chemical properties, and potential field of biomedical applications of SPIONs were also reviewed extensively [1].

The assessment of the role of SPIONs as a versatile platform for medical development requires more in-depth insight knowledge and constant study of relationships between nanoparticles size and size distribution, shape, magnetic properties and their biological application [1,22,23,24,25,26,27].

There are several established SPIONs synthesis methods, including co-precipitation, microemulsion, sol-gel, hydrothermal and thermal decomposition [2,7,8,9,12,13,14,15,16,18,20,22,24,25,28,29,30], each of those displaying advantages as well as drawbacks.

Our previous studies have addressed the role of pressure and temperature on the formation of hematite (Fe_2_O_3_) by the hydrothermal method [31,32].

In the case of high pressure hydrothermal synthesis, an external pressure higher than the water vapor pressure at equilibrium is used. Under these conditions, the quality of the obtained nanoparticles is remarkable because the solubility of inorganic materials increases with increasing pressure.

In the present paper, our aim is to control the synthesis parameters of nanostructured iron oxides in order to obtain magnetite nanoparticles in a single step, in environmentally friendly conditions, under inert gas atmosphere (external pressure).

Over the last decade, the concept of glioblastoma treatment was approached based on a mechanical transduction effect rather than a heating radiative effect for tumor cell death, an approach initiated by Kim et al. in 2010 [33], and recently reviewed by Naud et al. [34] (2020).

To this end, the hydrothermal synthesis of magnetite nanoparticles has been investigated, which offers numerous advantages over the use of commercial magnetite, such as: obtaining of crystalline nanoparticles in a single-step, no post-heat treatment is required for the synthesized particles, the process is relatively cost-effective (requires low energy consumption) and environmentally friendly (reaction takes place in an aqueous medium, without organic solvents), products with controllable size and good morphology [29,31]. This paper represents a step forward in demonstrating the potential of the hydrothermal process to obtain a variety of iron oxides and to control the synthesis parameters so as to obtain compounds with controlled morphology and composition.

The physico-chemical, structural, magnetic and biocompatible properties of magnetite prepared by hydrothermal method in different temperature and pressure conditions have been explored. Fourier-transform infrared (FT-IR) spectroscopy and X-ray diffraction (XRD) were used to identify the chemical bonds and main crystalline phases of the iron oxides. Microstructural characterization was carried out by transmission electron microscopy (TEM). The size distribution profile of magnetite nanoparticles in aqueous suspension was determined by Dynamic Light Scattering (DLS) technique. Vibrating-sample magnetometry (VSM) was used for magnetic characterizations. LDH and WST-1 tests were performed to evaluate the cytotoxicity of nanoparticles.

## 2. Materials and Methods

### 2.1. Synthesis

Magnetite was prepared by hydrothermal synthesis in a closed system, under inert gas atmosphere (Ar), starting from ferric chloride hexahydrate (FeCl_3_·6H_2_O), ferrous chloride tetrahydrate (FeCl_2_·4H_2_O), and NaOH of analytical grade. Reactants were purchased from Merck KGaA, Darmstadt, Germany. FeCl_3_·6H_2_O and FeCl_2_·4H_2_O were dissolved in distilled water and then sodium hydroxide was added dropwise until an alkaline pH was attained. As obtained precipitate was washed several times to remove secondary products and then transferred to the autoclave for 3 h of hydrothermal synthesis at 20–100 atm and 100–200 °C. Pressure is given by the inert gas purged above the precursor suspension. The resulted suspension was dried at −50 °C, using a freeze-dryer Martin Christ Alpha 1–2 LD plus (Martin Christ Gefriertrocknungsanlagen GmbH, Osterode am Harz, Germany). Experimental parameters of the investigated samples are presented in Table 1.

### 2.2. Characterization

Various characterization techniques like Fourier Transform Infrared spectroscopy (FTIR), powder X-Ray Diffraction (XRD), Transmission Electron Microscopy (TEM), Dynamic Light Scattering (DLS), and Vibrating Sample Magnetometry (VSM) were used to analyze the particles. FT-IR spectroscopy measurements were carried out with a ABB MB 3000 instrument (ABB Inc., Québec, QC, Canada) in the range of 4000–550 cm^−1^ using KBr pellets. XRD patterns were measured at 40 kV and 40 mA, using a BRUKER D8 ADVANCE diffractometer (Bruker AXS GmbH, Karlsruhe, Germany) with Cu Kα radiation source. XRD data was obtained from 4° to 74°, diffraction angles (2θ) using a step scan of 0.02°. TEM characterization was performed using a high-resolution transmission electron microscope 80–200 KV Titan THEMIS from Thermo Fisher (Former FEI, FEI Europe B.V., Eindhoven, Netherlands). The microscope was operated at 200 kV. Particle size distribution was performed by DLS technique, using Zetasizer Nano ZS 90 laser granulometer, Malvern Instruments (Worcestershire, UK), domain 0.6−3.0 µm, temperature range 20–90 °C, dispersion type wet, and Zetasizer software 7.02 (Malvern Instruments Ltd., Malvern Panalytical Ltd., Cambridge, UK). The magnetic measurements have been done with a MicroSense VSM magnetometer (MicroSense—A KLA Company, Lowell, MA, USA), at room temperature, up to 1 tesla. The toxicity of bare nanoparticles was assessed with HCT116 human colon cancer cells, using lactate dehydrogenase (LDH, Merck KGaA, Darmstadt, Germany) leakage test to assess cell membrane integrity and 4-[3-(4-iodophenyl)-2-(4-nitrophenyl)-2H-5-tetrazolio]-1,3-benzene disulfonate (WST-1, Roche Holding AG, Basel, Switzerland) test to assess cell metabolic activity. For the tests, HCT116 cells were seeded in 96-well plates at a density of 16 000 cells/well in 200 µL of culture medium and incubated for 24 h at 37 °C in a 5% CO_2_-humified atmosphere. Then, magnetic microparticles (MMP) were washed and dispersed in the culture medium at concentrations ranging from 3.9 mg/L to 250 mg/L. The supernatant of the cells was sampled for LDH assay, then replaced by 200 µL of these MMP suspensions. For dead cell control, 0.1% Triton-X100 was added to the cells. Cells were incubated for 24 h with the MMP.

For the LDH leakage test, 50 µL of the supernatant of each well were sampled and placed in a clean 96-well plate. LDH kit reagents were added in each well as recommended by the supplier and incubated at room temperature in the dark for 30 min. The reaction was stopped by 10 µL of 1 N HCl per well. The absorbance (Abs) was read at 490 nm with subtraction of the baseline signal at 690 nm. The percentage of LDH leakage was calculated as:

$$\%\;LDH\;Leakage=\frac{\left(Abs-Mean\left(Abs_{healthy\;cells}\right)\right)}{\left(Mean\left(Abs_{Triton}\right)-Mean\left(Abs_{healthy\;cells}\right)\right)}\times 100$$

For the WST-1 test, after the sampling for LDH test, the remaining supernatant was replaced by serum-free culture medium supplemented with 10% of WST-1 reagent and incubated 1 h at 37 °C in a 5% CO_2_-humified atmosphere. Then, to avoid interferences, 50 µL of supernatant of each well were sampled and placed in a new 96-well plate for absorbance reading at 450 nm with subtraction of the baseline signal at 650 nm. The percentage of viability was calculated as:

$$\%\;Viability=\frac{\left(Abs-Mean\left(Abs_{Triton}\right)\right)}{\left(Mean\left(Abs_{healthy\;cells}\right)-Mean\left(Abs_{Triton}\right)\right)}\times 100$$

## 3. Results

### 3.1. Fourier-Transform Infrared (FT-IR) Analysis

**Figure 1:**
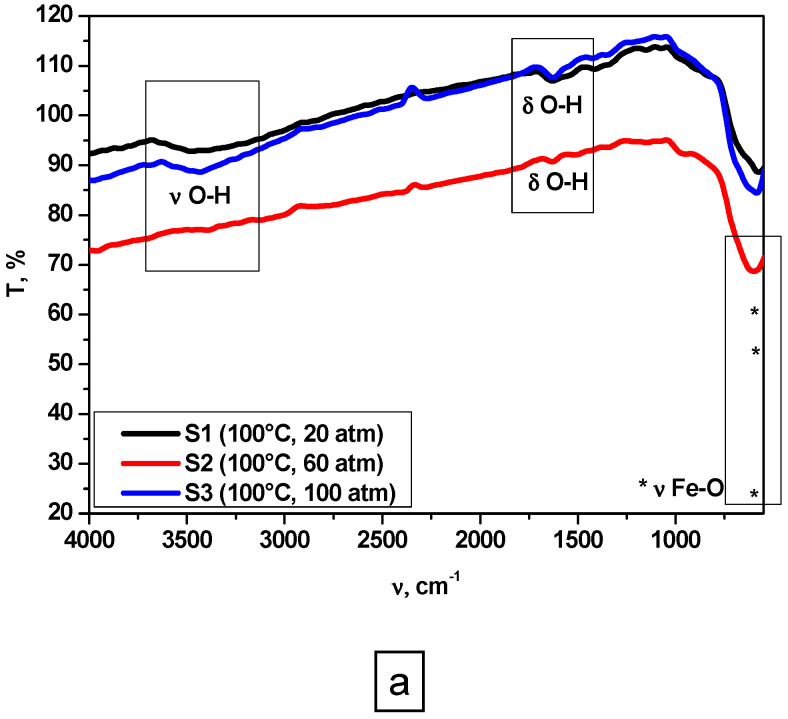

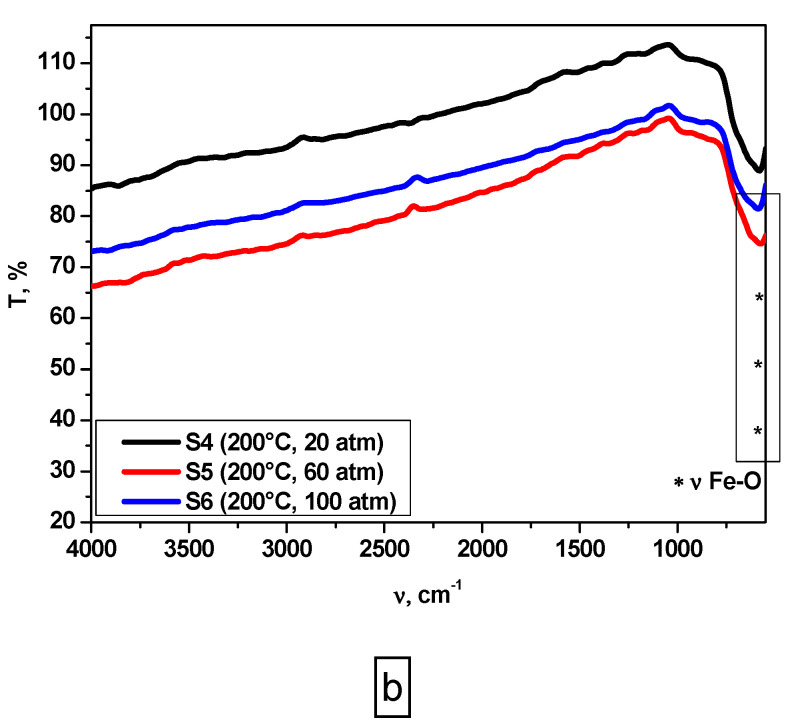
Fourier-transform infrared (FT-IR) analysis of samples prepared at: (**a**) 100 °C; (**b**) 200 °C.

**Figure 2:**
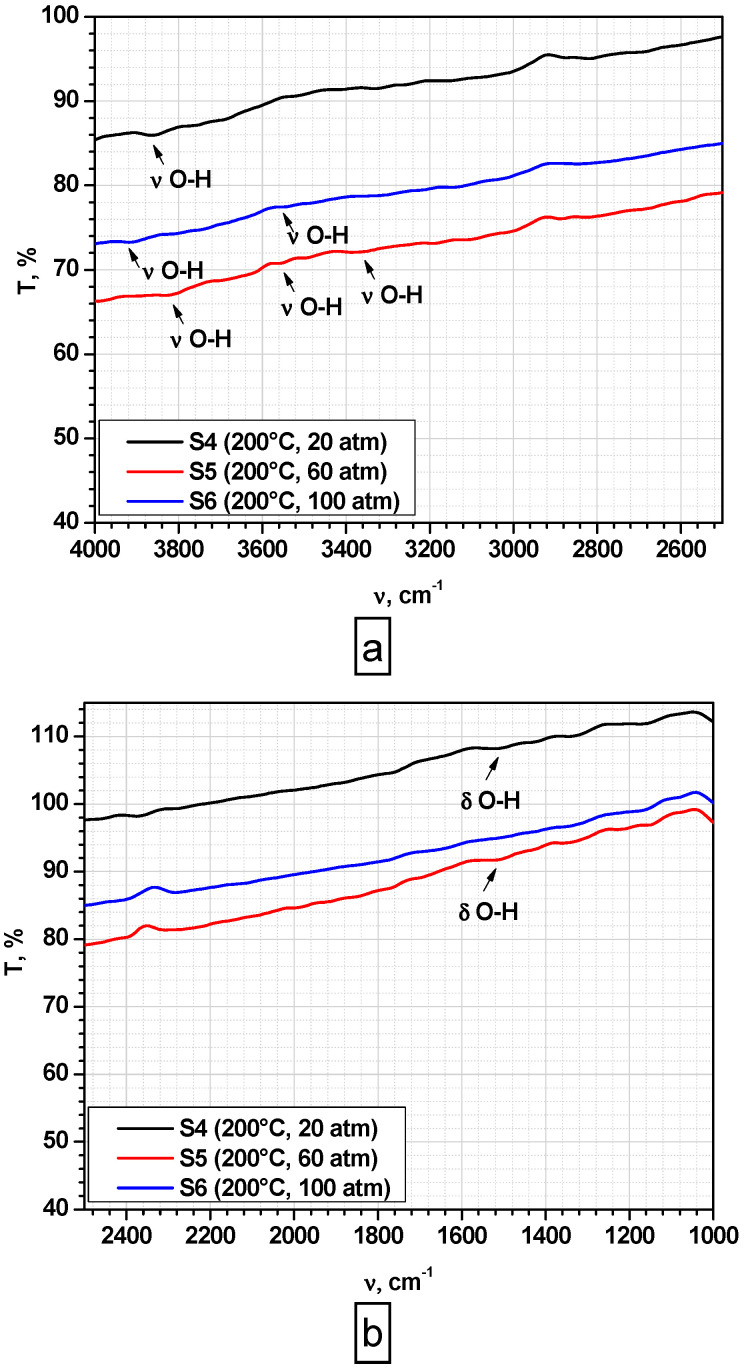
FT-IR analysis of samples prepared at 200 °C: (**a**) wavenumber range. 4000–2500 cm^−1^; (**b**) wavenumber range 2500–550 cm^−1.^

FT-IR spectroscopy analysis was carried out to obtain information regarding the chemical bonds of each sample. The spectra of the magnetite are shown in Figure 1 and Figure 2.

All samples exhibit similar absorption bands around 579–602 cm^−1^, which can be attributed to the Fe-O stretching vibration (Figure 1a,b), confirming magnetite presence [35,36,37]. In addition, weak absorption bands are observed at 1628 cm^−1^ (Figure 1a), in the case of samples prepared at 100 °C (S1–S3), which could be attributed to the bending mode of O-H bond or to the carbonate groups from atmospheric CO_2_ [35,36]. Additionally, all samples prepared at 100 °C (Figure 1a) exhibit similar absorption bands in the range of 3479–3394 cm^−1^, which can be attributed to hydroxyl groups (OH) related to the surface adsorbed water [38]. Low intensity peaks assigned to stretching (ν_O-H_) and bending (δ_O-H_) vibrations of OH groups could be observed in Figure 2, in the case of samples prepared at 200 °C (S4–S6). This behavior could be explained by the mechanism of formation of Fe_3_O_4_, described by Stoia et al. [39], combined with our previous findings regarding iron oxide’s behavior in high pressure conditions [38]:

Fe^2+^ + 2OH^−^ = Fe(OH)_2_ (1)

Fe^3+^ + 3OH^−^ = Fe(OH)_3_ (2)

Fe(OH)_3_ = FeO(OH) + H_2_O (3)

Fe(OH)_2_ + 2FeO(OH) = Fe_3_O_4_ + 2H_2_O (4)

According to this mechanism, Fe_3_O_4_ is formed as a result of the dehydration reaction of ferrous hydroxide and ferric oxyhydroxide (Equation (4)), the latter compound being produced by the partial transformation of Fe(OH)_3_ in high pressure conditions (Equation (3)) [40]. At the same time, the following reaction may occur:

Fe^2+^ + 2Fe^3+^ + 8OH^−^ = Fe_3_O_4_ + 4H_2_O (5)

The formation of FeO(OH) could explain the presence of OH groups in FT-IR spectra, especially in the case of samples synthesized at 100° C. Probably, reaction mechanism for magnetite formation is given by reactions (1)–(4), while in the case of samples synthesized at 200 °C, the reaction mechanism is given by reactions (1), (2), and (5).

### 3.2. X-ray Diffraction (XRD) Characterization

**Figure 3:**
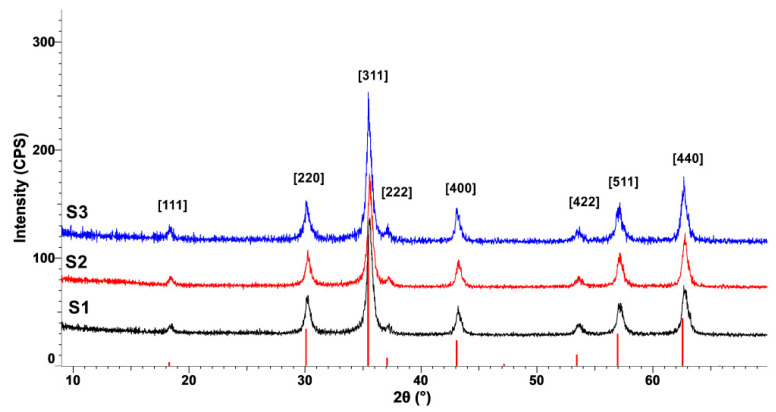
X-ray diffraction (XRD) patterns of samples synthesized at 100 °C.

**Figure 4:**
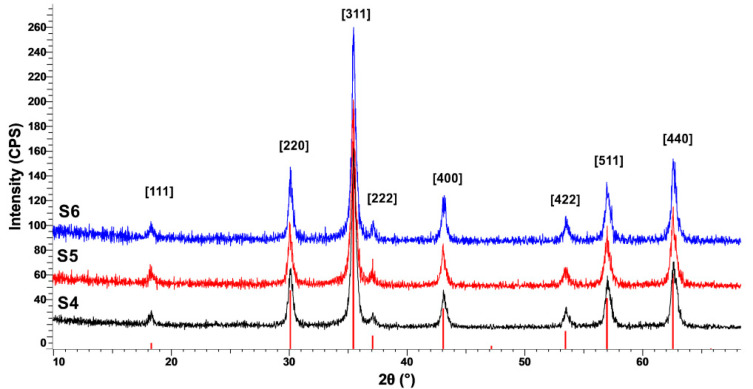
XRD patterns of samples synthesized at 200 °C.

The XRD patterns presented in Figure 3 and Figure 4 confirm the formation of a crystalline cubic structure with the peaks present at 2θ positions corresponding for the (111), (220), (311), (222), (400), (422), (511), and (440) hkl planes, associated to Fe_3_O_4_–Magnetite compound. The observable broadening of the diffraction peaks clearly indicates the fact that a nanosized crystalline magnetite formed. In the case of the samples synthesized at 100 °C (samples S1–S3, as presented in Table 1), the average size of the crystallites calculated for the (311) plane (D_(311)_) using Scherrer equation is around 20 nm. However, a tendency of increasing crystallite size with pressure increase can be observed: from 18.9 nm (sample S1, synthesized at 20 atm) to 22 nm (sample S3, synthesized at 100 atm). Samples synthesized at 200 °C (S4–S6, according to Table 1) also show an increase in crystallite size with increasing pressure: from 21.3 nm (sample S4, synthesized at 20 atm) to 26.8 nm (sample S6, synthesized at 100 atm). The crystallite size growth is influenced by both temperature and pressure increase.

### 3.3. Transmission Electron Microscopy (TEM) Characterization

**Figure 5:**
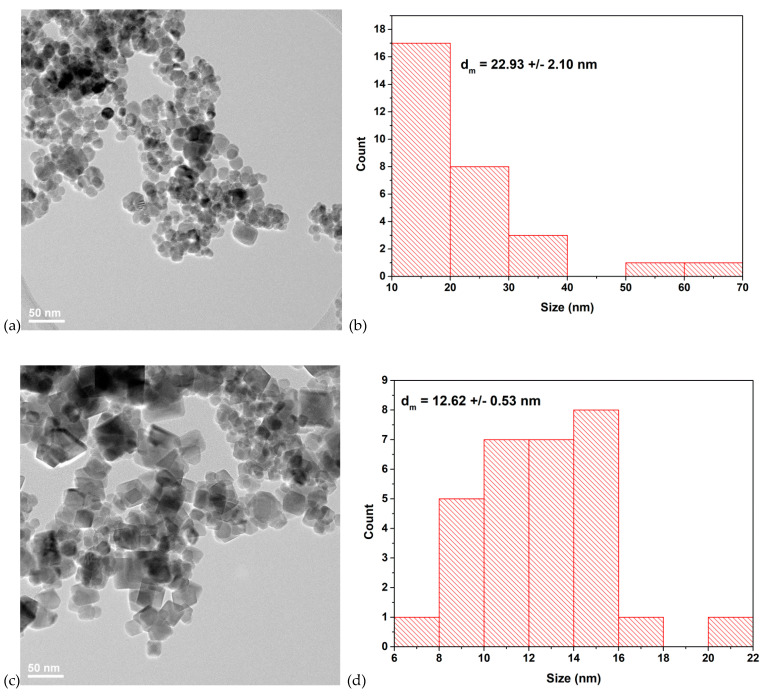
Transmission electron microscopy (TEM) images and core size distribution of samples: (**a**) S3 (prepared at 100 °C/100 atm); (**b**) size distribution of sample S3; (**c**) S6 (prepared at 200 °C/100 atm); (**d**) size distribution of sample S6.

Some examples of TEM micrographs of samples prepared at 100 atm (S3 and S6, see Table 1) are shown in Figure 5, showing a slightly different morphology at 100 and 200 °C, respectively. Thus, in the case of sample synthesized at 100 °C (S3), most particles are round shaped, but some rhombohedral particles can also be observed. Round shaped particles could be due to the presence of FeO(OH) phase. Sample S6 synthesized at 200 °C presents cubic/rhombohedral morphologies. These results are in accordance with the proposed reaction mechanism, showing that small amount of FeO(OH) could be present in samples prepared at 100 °C and 100 atm [41].

The size of hydrothermally synthesized magnetite nanoparticles is smaller than 50 nm, in accordance with the crystallite size calculated from the XRD analysis. Both samples exhibit moderate degrees of agglomeration, due to nano-scale sizes of hydrothermally synthesized materials.

### 3.4. Dynamic Light Scattering (DLS) Measurements

The determination of particle size using the Malvern Zetasizer ZS90 granulometer is based on a non-invasive technology, namely dynamic light scattering (DLS) emitted by a laser at a scattering angle of 90 degrees. This technique measures the diffusion of particles moving under Brownian motion and converts this to size and a size distribution using the Stokes-Einstein relationship.

Normally DLS is concerned with measurement of particles suspended within a liquid. The larger the particle, the slower the Brownian motion will be. The small particles will move faster than the large particles.

Velocity of the Brownian motion is defined by the translational diffusion coefficient (D), which can be converted into a particle size using the Stokes-Einstein equation:

d_H_ = kT/3ηπ D

where: d_H_ = hydrodynamic diameter, k = Boltzmann’s constant (1.38 × 10^−23^ NmK^−1^), T = absolute temperature (K), η = solvent viscosity (N·s·m^−2^), D = diffusion coefficient (m^2^·s^−1^).

The diameter that is measured in DLS is a value that refers to how a particle diffuses within a fluid so it is referred to as a hydrodynamic diameter. The diameter that is obtained by this technique is the diameter of a sphere that has the same translational diffusion coefficient as the particle. The translational diffusion coefficient will depend not only on the size of the particle core, but also on any surface structure, as well as the concentration and type of ions in the medium.

Sample preparation: aqueous dispersions stable in time (without sediments), transparent, with known optical properties (required for size measurement) were prepared using non-toxic polymeric dispersants such as polyacrylic acid or poly (acrylic acid sodium salt).

DLS has proven particularly popular due to its ability to provide information on both particle size and aggregation. The agglomeration tendency of nanosized particles is well known.

The results presented in Table 2 show that the hydrodynamic diameter of magnetite nanoparticles varies between 231 and 421 nm for samples prepared at 100 °C, respectively, between 517 and 692 nm for samples synthesized at 200 °C. An increase in hydrodynamic diameter can be observed with temperature increase (for samples prepared at the same pressure). It is likely that the surface structure changes with the increase of the synthesis temperature in hydrothermal conditions, which could explain an increase of the agglomeration degree and of the hydrodynamic diameter. Synthesis pressure influences the process of precipitation and crystallization [42].

### 3.5. Magnetic Measurements

**Figure 6:**
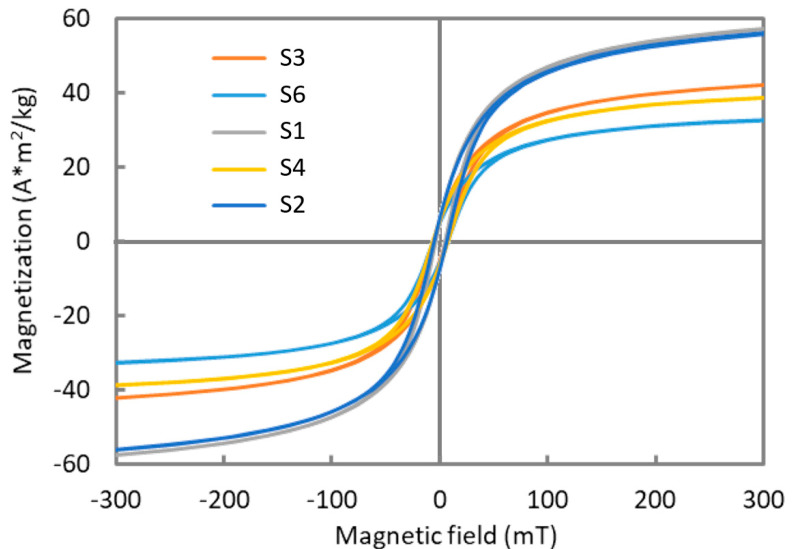
Room temperature magnetization of iron oxide particles.

The room temperature magnetization for different particles is shown in Figure 6. The magnetization curves show similar trends for all the particles, with low relative remanence (Mr/Ms), low coercive fields (Hc), and slowly saturating magnetization.

The mass magnetization at saturation (Ms) reaches 63 Am^2^/kg for particles synthesized at 100 °C and low pressure (S1-20 atm and S2-60 atm) but decreases down to 35 Am^2^/kg as the temperature and pressure increase. Although these values are lower than that of bulk magnetite (93 Am^2^/kg [43]), they agree well with measurement on magnetite particles with similar sizes, close to the superparamagnetic regime [44].

**Figure 7:**
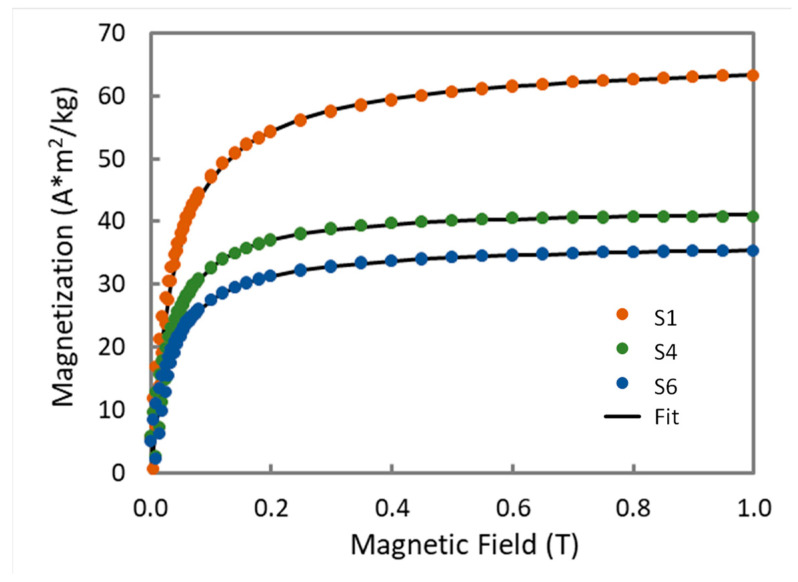
Langevin fit of the magnetization for some iron oxide particles.

The fitting of the magnetization curves also allows for an estimation of the average magnetic size. For an assembly of superparamagnetic particles, the measured magnetization is proportional to the Langevin function L(ξ):

$$L\left(\xi \right)=\coth\left(\xi \right)-\frac{1}{\xi }$$

with $\xi =m\mu_{0}H/k_{B}T$, where *m* is the particles magnetic moment, $\mu_{0}H$ is the magnetic induction, $k_{B}$ is the Boltzmann constant, and *T* is the temperature. The diameter ϕ for the particles is estimated using the *m* value deduced from the fit, with the equation $m\left(\phi \right)=\frac{\pi }{6}\phi^{3}\rho M_{s}$ where *ρ* and *M_s_* are the bulk magnetite density and the measured mass magnetization for each sample. If it is assumed that the size distribution for the particles follows a normal law distribution

$$P\left(\phi \right)=\frac{1}{\sqrt{2\pi }\sigma }e^{-\left(\frac{{\left(\phi -\phi_{0}\right)}^{2}}{2\sigma^{2}}\right)}$$

with P(ϕ) the density probability for diameter ϕ, with $\phi_{0}$ the average magnetic diameter and σ the standard deviation, the magnetization curve M(H) for the ensemble will be given by

$$M\left(H\right)=m_{T}\int_{0}^{\infty }L\left(H,m\left(\phi \right)\right)\;P\left(\phi \right)d\phi$$

with $m_{T}$ the total magnetic moment for the assembly. Examples of fits are shown in Figure 7 and the results are listed in Table 3.

From these results it can be observed that particles synthesized at 100 °C have nearly identical magnetic moments, at 20 × 10^3^ μ_B_, corresponding to magnetic cores of 10–11 nm, while the particles synthesized at 200 °C show slightly higher magnetic moments (25 × 10^3^ μ_B_) and larger magnetic cores (13 nm). It is nevertheless observed that the magnetic diameter is significantly smaller than the XRD crystallite size. The difference in the diameters varies from 8.5 nm (in S1) to 13,4 nm (in S6). Similar results have been reported for different types of magnetite particles, with diameters close to 20 nm, where the magnetic diameters are also close to 10 nm [45]. This observation is often attributed to the presence of a magnetic dead layer surrounding a magnetic core. However, when this layer is thick, as is the case here, such a simple picture is dubious, and the calculated dead layer can be considered an effective measure of the weakness of the magnetization.

In conclusion, the magnetic measurements indicate that the specific mass magnetization is higher with low temperature/low pressure synthesis, reflecting a less defective magnetic structure. As well, the Langevin analysis indicates that the magnetic core of particles synthesized at high temperature is larger, in accordance with the crystallite size analysis.

### 3.6. Toxicity Tests

**Figure 8:**
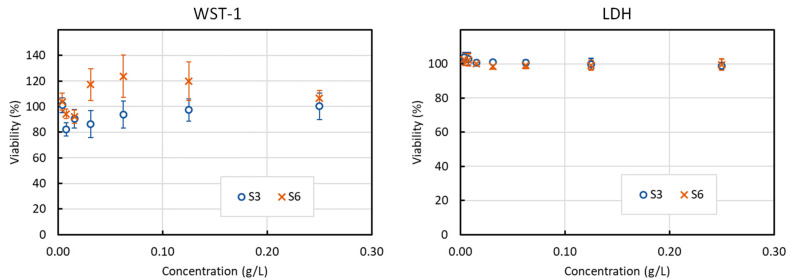
Viability of HCT116 cells after a 24 h incubation with S3 (100 atm/100 °C) or S6 (100 atm/200 °C) particles at concentration between 3,9 mg/L and 250 mg/L. Left: measured by WST-1 test; Right: measured by LDH test.

The toxicity of the particles was assessed by measuring the in vitro viability of HCT 116 human colon cancer cells incubated 24 h with increasing quantities of S3 and S6 samples. The results for the viability measured with LDH and WST-1 are presented in Figure 8. Despite some scatter in the WST-1 results, especially for low concentrations, it is observed that the particles show no intrinsic toxicity even at 0.25 g/L. These results are similar with those obtained with bare iron oxide particles, and this is a good indication that these particles could be suited for biomedical applications. It is nevertheless often observed that bare particles show less toxicity than surface-modified particles, and the absence of toxicity has yet to be demonstrated under in vivo conditions [46].

## 4. Conclusions

This study showed that our proposed hydrothermal method leads to nanostructured particles with good magnetic and biological properties (cytotoxic behavior). Good quality nanoparticles have been obtained in a single step, in a closed reaction system, in mild synthesis conditions (hydrothermal synthesis at 100 °C and pressure ≤ 100 atm), without the need of further thermal treatment. The novelty of the method consists in the fact that the pressure above the aqueous suspension in the autoclave corresponds to the vapor pressure of the substances in the reaction system, but also to the external pressure of the inert gas introduced.

The influence of pressure and temperature on particle size, magnetic, and cytotoxic behavior of magnetite prepared by hydrothermal synthesis was studied. It has been found that crystallite size increases with pressure and temperature increase, while hydrodynamic diameter is influenced by temperature. Magnetite formation has been proved in all samples by FT-IR analysis through the existence of Fe-O stretching vibration around 579–602 cm^−1^, and XRD characterization, through the formation of a crystalline cubic structure associated to Fe_3_O_4_—Magnetite compound. DLS measurements and TEM characterization showed a certain degree of agglomeration specific to nanoparticles. Additionally, cubic morphology typical for magnetite nanoparticles is more evident in the case of samples synthesized at 200 °C. The mechanism of magnetite formation is influenced by the synthesis temperature. At 100 °C, a secondary phase of FeO(OH) could be present in very small amounts.

In terms of magnetic measurements, it was found that particles synthesized at 100 °C have nearly identical magnetic moments, at 20 × 10^3^ μ_B_, corresponding to magnetic cores of 10–11 nm, while the particles synthesized at 200 °C show slightly higher magnetic moments (25 × 10^3^ μ_B_) and larger magnetic cores (13 nm). Additionally, the Langevin analysis indicated that the magnetic core of particles synthesized at high temperature is larger, in accordance with the crystallite size analysis.

The results for the viability measured with LDH and WST-1 tests revealed that the particles show only minor intrinsic toxicity even at a 250 mg/L dose, meaning that these particles could be suited for biomedical applications.

## Figures and Tables

**Table 1:** Synthesis conditions of magnetite nanoparticles prepared by hydrothermal synthesis.

Sample Name	Synthesis Conditions
S1	100 °C/20 atm
S2	100 °C/60 atm
S3	100 °C/100 atm
S4	200 °C/20 atm
S5	200 °C/60 atm
S6	200 °C/100 atm

**Table 2:** Mean particle size and median particle size, measured by Dynamic Light Scattering (DLS) method.

Sample Name	Synthesis Conditions	Mean Particle Size (Hydrodynamic Diameter), nm	Median Particle Size, nm	% of Median Sized Particles from All Size Values
S1	100 °C/20 atm	231	141.8	5.9
S2	100 °C/60 atm	421.4	396.1	24.3
S3	100 °C/100 atm	324.8	396.1	14.8
S4	200 °C/20 atm	517.5	531.2	26.1
S5	200 °C/60 atm	692	615.1	22.6
S6	200 °C/100 atm	539.6	615.1	20.4

**Table 3:** Magnetic properties of iron oxide particles.

Sample name	T	P	$\mu_{0}\mathrm{Hc}$	Mr/Ms	Ms	$m(\phi_{0})$	$\phi_{0}$	σ
	°C	atm	mT		Am^2^/kg	$\mu_{B}$	nm	nm
S1	100	20	4	0.09	63.3	20670	10.4	4.0
S2	100	60	6	0.11	62.6	20097	10.3	4.8
S3	100	100	5	0.11	46.0	20377	11.5	3.9
S4	200	20	7	0.15	40.8	24897	12.8	2.9
S6	200	100	7	0.15	35.2	24815	13.4	3.9
